# The unorthodox aquaporin-12L ortholog is essential for oogenesis in the crustacean *Artemia franciscana*

**DOI:** 10.1042/BSR20260496

**Published:** 2026-10-13

**Authors:** Ángel Rey-Alfonso, François Chauvigné, Roderick Nigel Finn, Joan Cerdà

**Affiliations:** 1 Institute of Marine Sciences, Spanish National Research Council (CSIC), 08003 Barcelona, Spain; 2 Institute of Biotechnology and Biomedicine (IBB), Universitat Autònoma de Barcelona, 08193 Bellaterra (Cerdanyola del Vallès), Spain; 3 Department of Biological Sciences, University of Bergen, 5006 Bergen, Norway

**Keywords:** Aqp12L, fertility, oogenesis, permeability, RNA interference

## Abstract

The intracellular unorthodox aquaporins, AQP11 and AQP12, remain the least characterized members of the aquaporin superfamily. Mammalian and teleost AQP12 channels have recently been established as polytransporters in native membranes. They are localized intracellularly in pancreatic acinar cells and considered to play roles in zymogen granule maturation and exocytosis. In contrast with the vertebrate channels, the permeability properties and physiological roles of invertebrate AQP12-like (Aqp12L) orthologs are completely unknown. Here, we report that the Aqp12L channel from the extremophile crustacean *Artemia franciscana* is specifically targeted to the membrane of the yolk platelets (YPs) when heterologously expressed in frog (*Xenopus laevis*) oocytes, as recently reported for the vertebrate orthologs. Using YPs isolated from *A. franciscana* Aqp12L-expressing frog oocytes, we further show that this channel can transport water, H_2_O_2_, ammonia, and urea but not glycerol. Immunostaining of the *A. franciscana* ovary using a specific Aqp12L antibody revealed that the *in vivo* localization of the channel also occurs in the YP membranes of vitellogenic oocytes. RNAi-mediated silencing of Aqp12L substantially reduces the offspring output of both ovoviviparous and oviparous females through the impairment of oocyte vitellogenesis (oocyte growth) and the premature activation of ovarian apoptotic and atretic pathways. These findings provide the first direct evidence demonstrating that invertebrate Aqp12L is a functional polytransporter with an essential role in crustacean oogenesis.

## Introduction

Aquaporins are small, hydrophobic integral membrane proteins that mediate the passive, osmotic gradient-driven transport of water and a range of other small uncharged solutes [1–3]. The gene superfamily is ubiquitous across all domains of life, with metazoan genomes encoding two major categories of orthodox and unorthodox channels. The former orthodox repertoires are phylogenetically subdivided into classical (e.g. AQP4-related), aquaglyceroporin (e.g. AQP3-related), and AQP8-type channels, while the latter are represented by AQP11 and AQP12-type channels [4–7].

Amongst all aquaporins, AQP12 remains one of the least characterized. Studies in mammals, however, suggest that this intracellular channel is involved in endoplasmic reticulum (ER) homeostasis, as well as in the maturation and exocytosis of zymogen granules (ZG) of pancreatic acinar cells [8–11]. Most recently, a highly conserved *in vivo* localization of the AQP12-type channels was revealed in the yolk platelet membranes (YPMs) of developing vertebrate and invertebrate oocytes and embryos [11]. Yolk platelets (YPs) are low-permeable membrane-bound intracellular vesicles that form during vitellogenesis (oocyte growth) in metazoans to store vitellogenin-derived yolk proteins in both liquid and “crystalline-like” structures [12,13]. The specific targeting of AQP12 to the YPM in frog (*Xenopus laevis*) oocytes was leveraged to decipher the biophysical properties of the vertebrate channels in native membranes, revealing that AQP12 channels are polytransporters permeable to water, H_2_O_2_, ammonia, and urea, but not glycerol [11]. While these studies shed light on the potential role of AQP12 in vertebrate systems, its permeability properties and function in invertebrates remain largely unexplored.

Within arthropod genomes, AQP12-like (Aqp12L) channels are commonly present as single-copy genes, although in certain lineages, including some arachnids, branchiopods, and copepods, multiple copies may exist [3,6,14,15]. Due to the absence of a unified nomenclature, the channels are also referred to as superaquaporins and Aqp11-like channels [10,16,17]. In crustaceans, only limited evidence exists for the possible functional role of the channel. In the barnacle *Balanus improvisus, in silico* structural analyses of Aqp12L suggest that the protein might not facilitate the transport of water or any other solutes, raising the possibility of a non-canonical or vestigial function of the channel [18]. In decapods, such as the Pacific white shrimp, *Litopenaeus vannamei*, and Chinese mitten crab, *Eriocheir sinensis*, the channels are considered important for osmotic balance via ER-dependent signaling, molting coordination, and muscle matrix stabilization [16,17]. However, for the parasitic copepod salmon louse, *Lepeophtheirus salmonis*, specific Aqp12L antibodies show that the proteins are localized in the intracellular YPs [6,11].

The brine shrimp *Artemia franciscana* has a single *aqp12L* gene [15], thus offering a suitable model to investigate Aqp12L function in crustaceans. Under favorable environmental conditions, this species reproduces ovoviviparously via internal fertilization, with embryos hatching inside the female for release as free-swimming nauplii. When adverse environmental conditions predominate, such as extreme salinity, elevated temperatures, short photoperiod, or hypoxia, reproduction shifts to an oviparous mode, leading to the release of encysted dormant embryos (cysts) [19–21]. The dual reproductive strategies of *A. franciscana*, single-copy *aqp12L* gene, and earlier evidence of Aqp12L YP localization in the salmon louse [6] make this species an ideal model for investigating the transport properties and functional role of this intracellular channel in invertebrates.

## Hypothesis

We hypothesize that the *A. franciscana* Aqp12L paralog is a functional channel also localized in the YPM of oocytes, where it can play a role during yolk formation. To test this hypothesis, we determined the permeability properties of Aqp12L and investigated its functional role during both ovoviviparous and oviparous development by combining mRNA and protein expression profiling and RNA interference (RNAi)-mediated gene silencing.

## Results

### *A. franciscana* Aqp12L is a polytransporter specifically targeted to the oocyte YPM of *X. laevis* oocytes

To assess the subcellular localization of *A. franciscana* Aqp12L and its permeability properties, a cDNA encoding the channel tagged with human influenza hemagglutinin (HA) was cloned, and the corresponding cRNA was expressed in *X. laevis* oocytes. The subcellular localization of Aqp12L-HA was evaluated via immunofluorescence microscopy on oocyte sections and isolated YPs with protein expression also confirmed by immunoblotting. This confirmed that the *A. franciscana* Aqp12L-HA remains intracellular and targeted to the YPM (Figure 1A–C).

**Figure 1:**
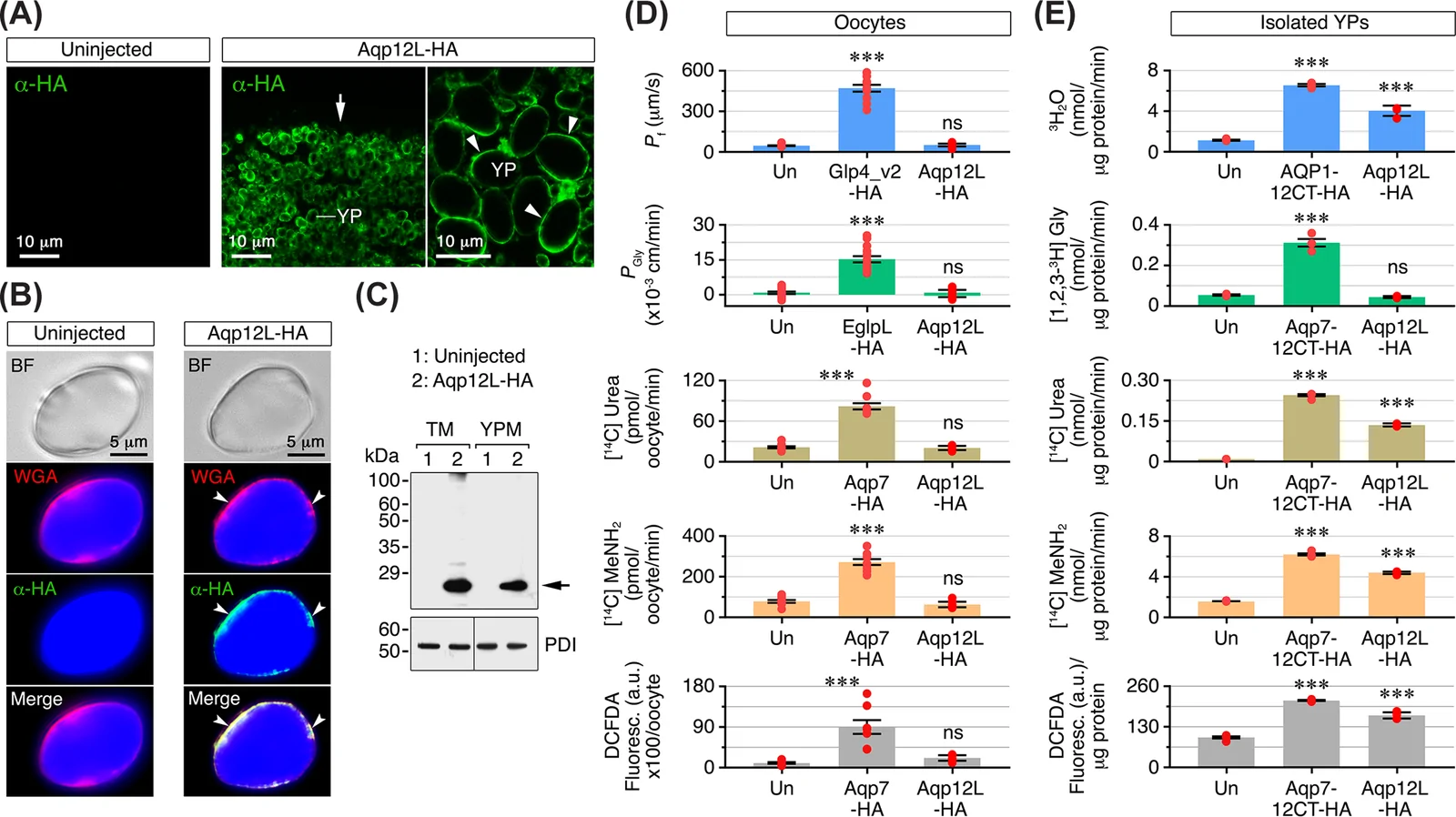
The *A. franciscana* Aqp12L is a polytransporter specifically targeted to the YPM of *X. laevis* oocytes (**A**) Representative immunostaining of HA-tagged Aqp12L in *X. laevis* uninjected oocytes (control) and oocytes expressing the channel using an anti-HA antibody. The arrow points at the plasma membrane, whereas the arrowhead indicates the YPM. YP, yolk platelet. (**B**) Immunolocalization of Aqp12L in YPs isolated from control and Aqp12L-HA-expressing oocytes. The YPM (arrowheads) is counterstained with fluorescent-conjugated wheat germ agglutinin (WGA; red), whereas the yolk proteins inside the YPs are labeled with an anti-vitellogenin antibody (blue). (**C**) Immunoblot of protein extracts from total membrane (TM) and YPM of uninjected oocytes or oocytes expressing Aqp12L-HA using the antibody specified above. The protein disulfide-isomerase (PDI) was used as a protein loading control. The arrows indicate the aquaporin monomers. Molecular mass markers (kDa) are on the left. Uncropped blots are shown in Supplementary Figure S5. Water and solute (glycerol, urea, MeNH_2_, and H_2_O_2_) permeability of *X. laevis* uninjected oocytes (Un; controls) and oocytes expressing Aqp12L-HA (**D**), as well as of YPs extracted from control oocytes and Aqp12L-HA-injected oocytes (**E**). Oocytes expressing HA-tagged *A. franciscana* Glp4_v2 and EglpL, zebrafish Aqp7 (Aqp7) and Aqp7-12CT-HA, and human AQP1-12CT-HA chimeric constructs, were used as positive controls for water and solute transport. In panels (D and E), data (mean ± SEM, *n* = 12–17 oocytes from two females in panel (D) and *n* = 4 different pools of oocyte YPs collected from two females in panel (E), red dots) were statistically analyzed by the unpaired Student’s *t*-test ***P < 0.001; with respect to the controls. ns, not statistically significant.

Channel-facilitated permeability to water, glycerol, urea, ammonia, and hydrogen peroxide (H_2_O_2_) across the plasma membrane and isolated YPs of uninjected (control) oocytes and oocytes expressing Aqp12L was subsequently determined volumetrically or using radioactive or fluorescent substrate uptake assays. As positive controls, we employed HA-tagged plasma membrane-targeted channels, such as *A. franciscana* Glp4_v2 and EglpL [15], and zebrafish wild-type Aqp7 (Aqp7) [22], and YP membrane-directed chimeric constructs, such as zebrafish Aqp7 carrying the C-terminus of Aqp12 (Aqp7-12CT) and the human AQP1 carrying the C-terminus of AQP12 (AQP1-12CT) [11]. Oocytes expressing Glp4_v2-HA or EglpL-HA elicited increased osmotic water and glycerol permeabilities (*P*_f_ and *P*_gly_, respectively) compared with the control oocytes, while those expressing Aqp7-HA were permeable to urea, the ammonia analogue methylamine (MeNH_2_), and H_2_O_2_ (Figure 1D). In contrast, oocytes injected with Aqp12L-HA, which remained intracellular, did not permeate water or any of the solutes tested (Figure 1D). The YPs isolated from oocytes expressing AQP1-12CT-HA or Aqp7-12CT-HA showed increased water, glycerol, urea, MeNH_2_, and H_2_O_2_ uptake, respectively, compared with the YPs collected from control oocytes (Figure 1E). Similarly, Aqp12L-inserted YPs were more permeable to water, urea, MeNH_2_, and H_2_O_2_, but not glycerol (Figure 1E). These functional assays therefore indicate that *A. franciscana* Aqp12L is a functional polytransporter with similar permeability properties to the vertebrate orthologs.

### Aqp12L expression pattern during ovoviviparous and oviparous development

To investigate the role of Aqp12L during *A. franciscana* ovoviviparous and oviparous reproductive development, we first investigated the pattern of expression of the channel at the mRNA and protein level across key developmental stages by quantitative reverse transcription polymerase chain reaction (RT-qPCR) and immunoblotting, respectively. For the ovoviviparous mode, we examined female gonads containing the ovary and ovisacs holding embryos destined to hatch free-swimming nauplii, as well as the nauplii themselves. For the oviparous mode, we analysed female gonads (ovary plus ovisac) producing encysted diapause embryos as well as the released dormant cysts. The RT-qPCR data revealed unchanged *aqp12L* mRNA levels in nauplii-destined gonads, free-swimming nauplii and cyst-destined gonads (Figure 2A). However, a marked reduction in the accumulation of the *aqp12L* transcripts was observed in released cysts (Figure 2A).

**Figure 2:**
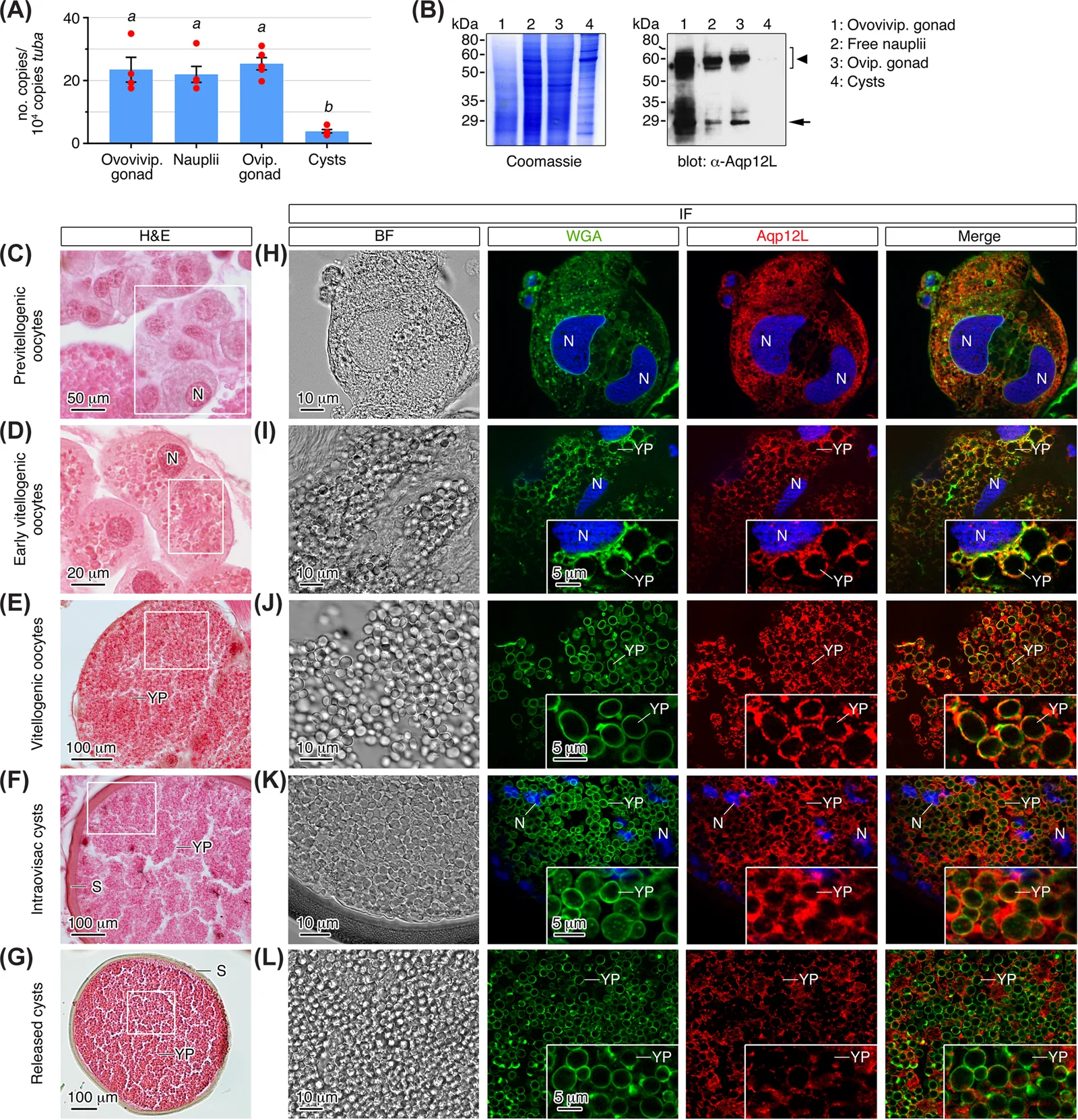
Expression pattern and subcellular localization of *A. franciscana* Aqp12L during ovoviviparous and oviparous development (**A**) Expression levels of aqp12L mRNA in female gonads destined to produce nauplii (ovoviviparous reproduction), free nauplii, gonads producing diapause cysts (oviparous reproduction), and free diapause cysts as determined by RT-qPCR. Transcript levels of α-tubulin (tuba) were used as reference. Data (mean ± SEM; *n* = 4–5 independent experiments, red dots) were statistically analyzed by one-way ANOVA followed by the Tukey’s multiple comparisons test. Bars with different superscripts are significantly different (*P* < 0.001). (**B**) Coomassie Blue staining (left) and immunoblot (right) of protein extracts from the same developmental stages shown in panel (A) probed with the *A. franciscana*-specific α-Aqp12L antibody. The arrow in the blot points to the immunoreactive band that closely matches the predicted monomeric molecular mass of Aqp12L (28.4 kDa), whereas the arrowhead indicates potential post-translational modifications and/or oligomerization of the channel. Molecular markers (kDa) are on the left. Uncropped blot is shown in Supplementary Figure S5. (**C**–**G**) Paraffin sections of ovaries and cysts stained with hematoxylin and eosin (H&E) showing oocytes at different vitellogenic stages and intraovarian and released cysts as indicated. (**H**–**L**) Immunofluorescence microscopy (IF) of the same stages shown in (C–G), showing bright field (BF) and epifluorescence images of Aqp12L localization (red) in areas marked with white squares in panels (C–G). Sections were counterstained with 4′,6-diamidino-2-phenylindole (DAPI; blue) and fluorophore-coupled lectin wheat germ agglutinin (WGA; green) to stain the YP membrane. Merged images are shown to the right. Control sections incubated with the preabsorbed antiserum were negative (Supplementary Figure S2). N, nuclei; YP, yolk platelet; S, shell.

For Aqp12L protein detection, we produced an *A. franciscana* Aqp12L-specific rabbit antibody, which was validated by immunoblotting of *X. laevis* oocytes expressing Aqp12L, and of protein extracts from ovoviviparous and oviparous gonads (Supplementary Figure S1). In *X. laevis* oocytes, the anti-Aqp12L antibody detected a band below the 29-kDa marker, consistent with the predicted molecular mass of the *A. franciscana* Aqp12L monomer (28.4 kDa) (Supplementary Figure S1B). In the gonad extracts, the ∼29-kDa band was also detected, as well as an additional band of ∼60 kDa (Supplementary Figure S1C). This additional reactive band may correspond to post-translational modifications of Aqp12L and/or oligomerization of the channel, since we could not reveal any signal in membranes incubated with the preadsorbed antiserum (Supplementary Figure S1C). Subsequent immunoblotting of protein extracts from the same developmental stages used for the RT-qPCR analysis revealed Aqp12L polypeptides in the gonad and nauplii, but not in released diapause cysts (Figure 2B), consistent with the mRNA reduction. Together, these data indicate that Aqp12L is transcribed and translated during oogenesis and embryogenesis in both reproductive modes, but virtually ceases in the final stages of oviparous cyst formation.

The cellular localization of the Aqp12L channel was examined in previtellogenic, early vitellogenic, vitellogenic oocytes, and intra-gonadal and released cysts using immunofluorescence microscopy and the Aqp12L antibody (Figure 2C–G). Immunostaining revealed that in previtellogenic oocytes, devoid of YPs, Aqp12L was distributed in the cytoplasm (Figure 2H). In contrast, Aqp12L was detected in the cytoplasm and the YPM in early vitellogenic and vitellogenic oocytes, and intra-gonadal cysts (Figure 2I–K). However, in released diapause cysts Aqp12L was no longer detected in the YPM, with only some residual staining noted in the cytoplasm (Figure 2L). In all stages, the specificities of the immunoreactions were demonstrated by the lack of staining when the antisurum was preincubated with the antigenic peptide (Supplementary Figure S2). These results suggest that in *A. franciscana*, Aqp12L is recruited to the YPM specifically at the onset of vitellogenesis and remains integrally localized throughout oocyte growth and early embryonic development.

### RNAi-mediated Aqp12L knockdown impairs ovoviviparous and oviparous development

To investigate the physiological role of Aqp12L in *A. franciscana* during ovoviviparous and oviparous development, we performed dsRNA-mediated RNAi to knockdown *aqp12L* expression. We initially used a dsRNA that covered the middle region of the coding sequence (CDS) of the Aqp12L mRNA (nucleotides 270–691) (Supplementary Table S1). Females with gonads destined for either reproductive mode were injected with dsRNA targeting *aqp12L* (dsAqp12 RNA) or with a control dsGFP RNA. Female survival at 8 days postinjection (DPI) was similar between the two groups (∼80%) (Supplementary Figure S3), where the RT-qPCR analysis confirmed a strong reduction of *aqp12L* mRNA levels in ovoviviparous gonads at 5 DPI and free nauplii following dsAqp12 RNA treatment (∼92% and ∼75% reduction, respectively, compared with the controls) (Figure 3A). Western blot analysis revealed undetectable Aqp12L protein levels in ovoviviparous gonads from females treated with dsAqp12L RNA, with Coomassie Blue staining performed in parallel to ensure equal protein loading (Figure 3B). The marked reduction in Aqp12L mRNA and protein in the gonads from females injected with dsAqp12L RNA was associated with a 63% decrease of the offspring output compared with controls (Figure 3C). In oviparous females treated with dsAqp12L RNA, *aqp12L* mRNA expression was reduced by ∼90% in both gonads and cysts compared with controls (Figure 3D), while immunoblotting showed a marked reduction of the Aqp12L protein (Figure 3E). In this case, however, the offspring production was reduced by ∼87% compared with controls (Figure 3F). The same results were obtained using a different dsAqp12L RNA covering the 5′ end of the CDS of the Aqp12L mRNA (nucleotides 6–268) (Supplementary Table S1 and Supplementary Figure S4).

**Figure 3:**
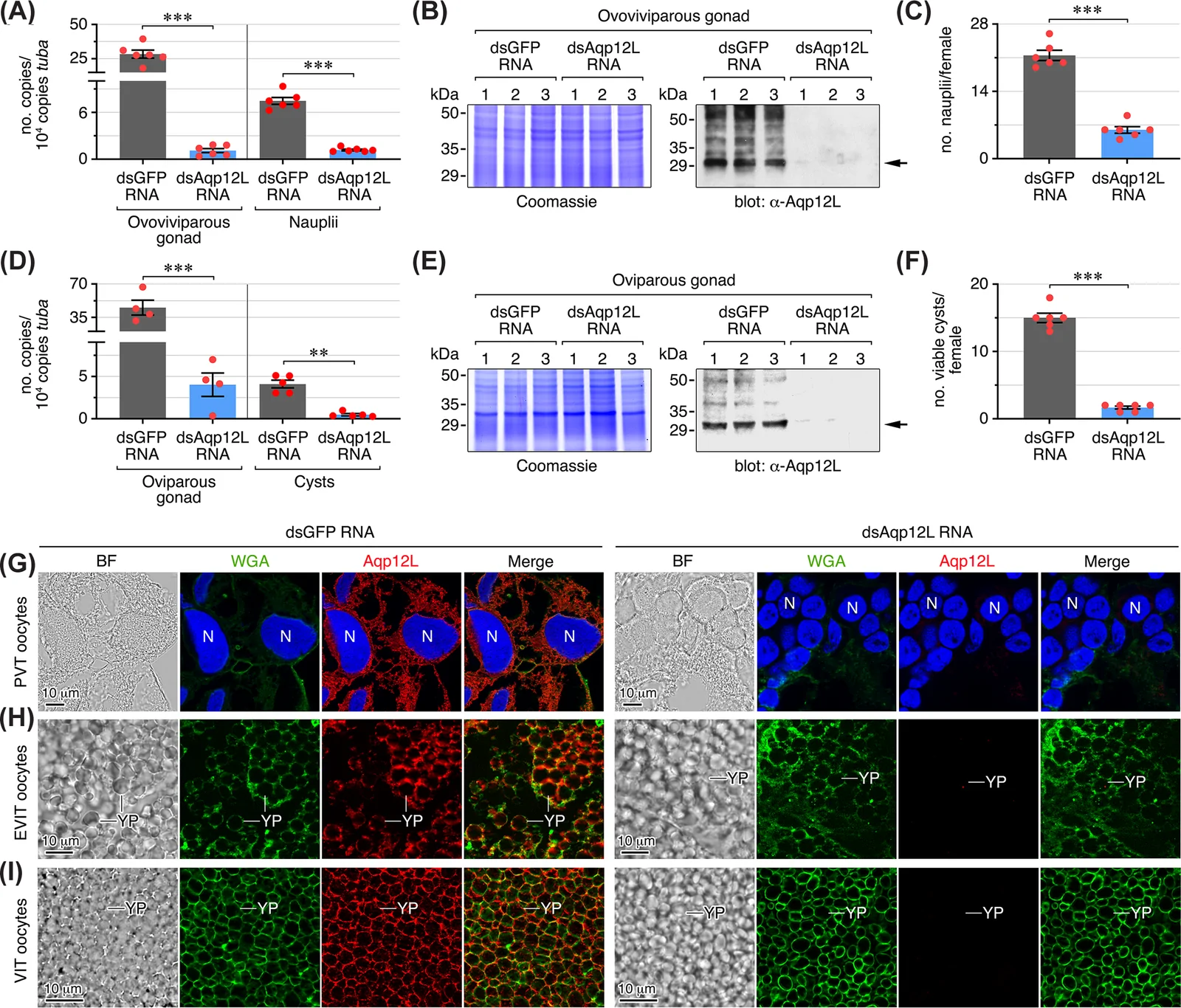
RNAi-mediated Aqp12L knockdown impairs ovoviviparous and oviparous reproduction Decrease of *aqp12L* transcript levels in gonads, nauplii, and released cysts from ovoviviparous (**A**) or oviparous (**D**) females injected with dsGFP (control) or dsAqp12L RNAs determined by RT-qPCR. Data (mean ± SEM; *n* = 4–6 independent experiments, red dots) from each developmental stage were statistically analyzed by an unpaired Student’s *t*-test (**, *P* < 0.01; ***, *P* < 0.001; with respect to the controls). Coomassie Blue staining (left) and Aqp12L immunoblots (right) of protein extracts from ovoviviparous (**B**) or oviparous (**E**) gonads (*n* = 3 females/treatment). Arrows in each blot point to the Aqp12L monomer, whereas the molecular markers (kDa) are shown to the left. Uncropped gels and blots are shown in Supplementary Figure S5. Production of nauplii (**C**) or diapause cysts (**F**) by females treated with dsGFP or dsAqp12L RNAs. In each panel, data (mean ± SEM; *n* = 6 independent experiments, red dots) were statistically analyzed by an unpaired Student’s *t*-test (***, *P* < 0.001; with respect to the controls). (**G**–**I**) Immunolocalization of Aqp12L in previtellogenic (PVT), early vitellogenic (EVIT), and vitellogenic (VIT) oocytes in the ovary of ovoviviparous females injected with dsGFP or dsAqp12L RNAs. BF and Aqp12L (red), WGA (green) and DAPI (blue) epifluorescence images, and merged images, are shown. N, nuclei; YP, yolk platelets.

To further confirm the efficiency of the RNAi-mediated *aqp12L* knockdown, immunostaining was carried out on ovaries from ovoviviparous females injected with either dsGFP or dsAqp12L RNAs. In dsGFP RNA-injected controls, Aqp12L was detected in the cytoplasm of previtellogenic oocytes (Figure 3G), and in the YPM of early vitellogenic and vitellogenic oocytes (Figure 3H,I). In contrast, the ovaries from dsAqp12L RNA-treated females showed a complete loss of Aqp12L immunostaining across all oocyte stages (Figure 3G–I). This indicates that dsRNA injection significantly reduces both Aqp12L mRNA and protein levels during ovoviviparous and oviparous development, leading to an impaired offspring production.

### Aqp12L is required for oocyte growth during *A. franciscana* oogenesis

To evaluate the impact of *aqp12L* silencing on oocyte development and offspring reduction, we performed a phenotypic analysis of the ovary from females injected with either dsGFP or dsAqp12L RNAs at 3 and 5 DPI using stereomicroscopy and light microscopy of hematoxylin and eosin-stained tissue sections. In dsAqp12L-treated females, oocyte numbers in the ovary as well as number of embryos within the ovisac were apparently reduced compared with controls at both sampling days (Figure 4A,B). Quantification of the percentage of previtellogenic and vitellogenic oocytes in the ovary from females treated with dsGFP or dsAqp12L RNAs indicated that at 3 DPI the ovaries from the dsGFP RNA females contained ∼30% of previtellogenic and ∼70% of vitellogenic oocytes, while the ovaries from the dsAqp12L females showed a reversal of this ratio, with ∼70% and ∼30% of previtellogenic vitellogenic oocytes, respectively (Figure 4C). At 5 DPI, this shift was more pronounced, with the dsAqp12L RNA ovaries containing ∼80% of previtellogenic and only ∼20% of vitellogenic oocytes, compared with stable proportions in the controls (Figure 4C). These data indicate that Aqp12L silencing markedly compromises vitellogenesis (oocyte growth) in *A. franciscana*.

**Figure 4:**
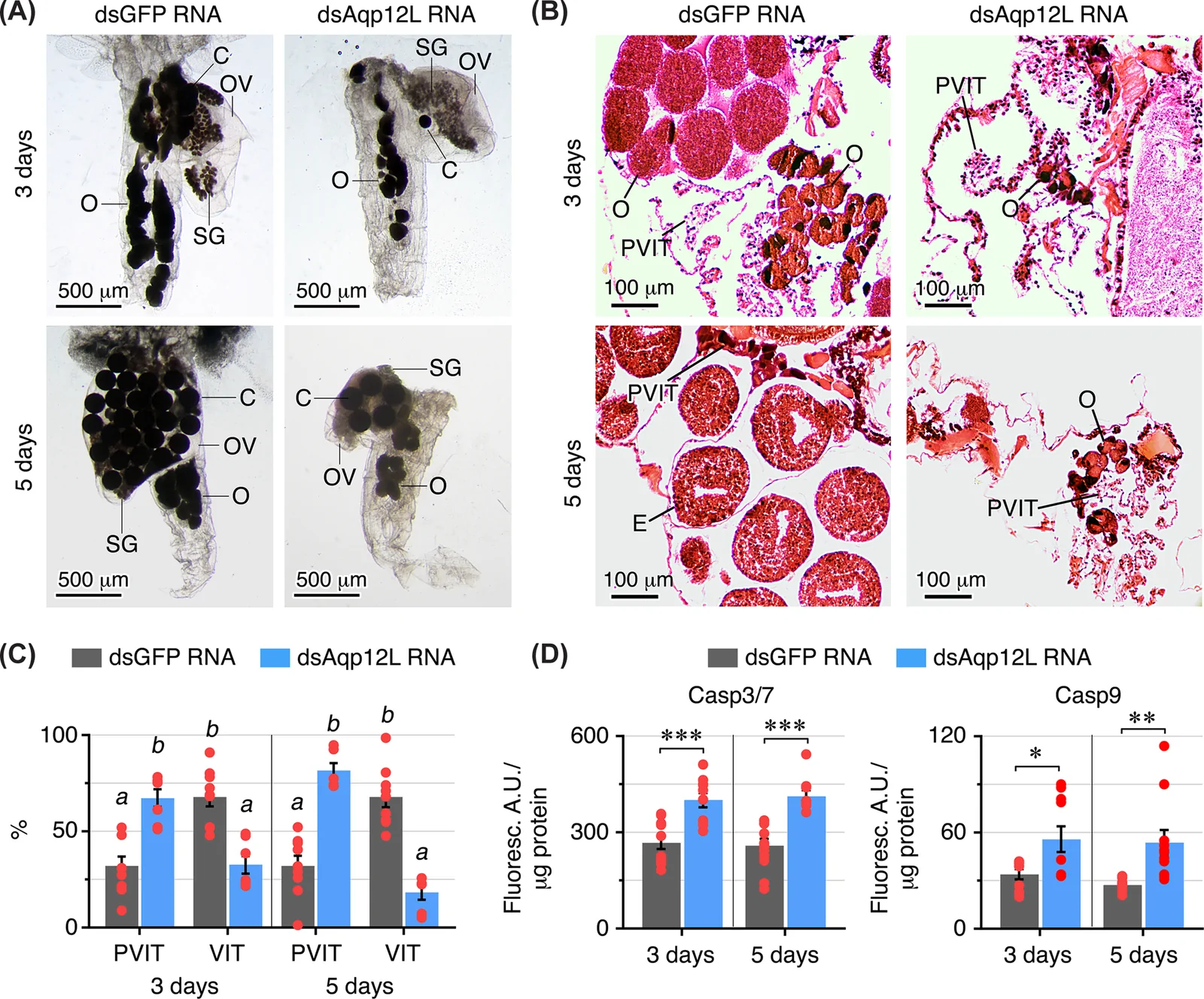
*A. franciscana* Aqp12L is required for oocyte growth Photomicrographs (**A**) and paraffin sections stained with hematoxylin and eosin (**B**) of the gonads from females treated with dsGFP (control) or dsAqp12L RNAs at 3 and 5 days post injection. O, oocyte; OV, ovisac; PVIT, previtellogenic oocyte; E, embryo; C, cyst; SG, shell gland. (**C**) Percentage of PVIT and vitellogenic (VIT) oocytes in the ovary of females from both groups. Data (mean ± SEM; *n* = 6–9 females, red dots) were statistically analyzed by one-way ANOVA followed by the Tukey’s multiple comparisons test. Bars with different superscripts are significantly different (*P* < 0.01). (**D**) Caspase-3, -7, and -9 (Casp3/7 and Casp9) activities in the ovary from dsGFP and dsAqp12L RNA-treated females. Data (mean ± SEM; *n* = 8–12 independent experiments, red dots) were statistically analyzed by an unpaired Student’s t-test (*, *P* < 0.05; **, *P* < 0,01; ***, *P* < 0.001, as indicated in brackets).

To further investigate whether the absence of Aqp12L could induce oocyte apoptosis and atresia in the *A. franciscana* ovary, the ovarian activities of caspase-3/7 and caspase-9 were determined at 3 and 5 DPI of dsGFP and dsAqp12L RNA treatments. The results showed an ∼35% and ∼44% increase in caspase-3/7 and caspase-9 activities, respectively, in the ovaries of dsAqp12L RNA-treated females compared with the dsGFP RNA controls at both 3 and 5 DPI (Figure 4D). Our data therefore suggest that the offspring reduction of the dsAqp12L RNA-treated females likely results from impaired oocyte growth caused by activated apoptotic mechanisms.

## Discussion

The present study shows that the *A. franciscana* Aqp12L channel is targeted to the intracellular YPM of developing oocytes *in vivo* regardless of the reproductive mode, as well as *ex vivo* when heterologously expressed in frog oocytes. These observations confirm that the YPM is an important intracellular site for AQP12 function in metazoan organisms [11].

The functional assays comparing the transport properties of the Aqp12L channel expressed in frog oocytes and isolated YPs demonstrated that water and solute passage only occurs in the vesicle-expressing system. This is consistent with the absence of the channel in the frog oocyte plasma membrane and localization in the YPM. It is further validated by the control channels Glp4_v2, EglpL, Aqp7, and AQP1, which show water and solute transport when localized in the plasma membrane or in the YPM when expressed as chimeric constructs bearing the AQP12 or Aqp12 C-terminus. The data for the Aqp12L-expressing YPs thus show that the channel is a polytransporter, capable of permeating water, H_2_O_2_, ammonia, and urea, but not glycerol, as previously observed for the vertebrate channels [11]. These findings therefore suggest that the transport properties are conserved between the vertebrate and invertebrate AQP12-related channels.

Due to the dual reproductive strategy of *A. franciscana*, switching from ovoviviparity to oviparity, we analyzed Aqp12L expression during both reproductive modes. The results indicate that Aqp12L is equally expressed at the mRNA and protein levels in the gonads of both ovoviviparous and oviparous females. However, while Aqp12L persists in nauplii, it is markedly reduced in released diapause cysts. Furthermore, immunofluorescence microscopy using a membrane marker revealed that endogenous *A. franciscana* Aqp12L is localized to the YPM of vitellogenic oocytes, either destined to produce nauplii or diapause cysts, as well as of developing cysts inside the female. These findings are therefore consistent with the YP localization of Aqp12L in salmon louse oocytes [6,11]. Upon release of the diapause cysts into the external environment, Aqp12L is no longer detectable in the YPM. These resistant cysts exhibit several adaptations, including the development of a protective shell, the accumulation of trehalose and glycerol, and the expression of late embryogenesis abundant (LEA) proteins and molecular chaperones [15,23–29]. Such adaptations confer resistance of embryos to extreme stressors such as UV radiation, temperature fluctuations, and desiccation [30]. It therefore appears that the expression of Aqp12L in the YPM is temporally restricted to the stages of oocyte growth and embryogenesis, suggesting a developmental function more closely aligned with oogenesis rather than during stress tolerance.

To investigate the physiological role of Aqp12L, we performed RNAi-mediated *aqp12L* gene silencing using the same methods as those employed previously for heat shock proteins and LEA proteins [31–34], as well as aquaglyceroporins and trehalose transporters [15,29]. In the present work, we successfully knocked down *aqp12L* expression in the gonads after five days of a single dsRNA injection, suggesting an apparent long half-life of the protein. Nevertheless, RNAi was able to efficiently reduce the mRNA and protein levels of Aqp12L in the ovary of females undergoing either ovoviviparous or oviparous reproduction. This caused an impairment of the offspring production, for both nauplii and cyst outputs, highlighting a crucial role for Aqp12L across both ovoviviparous and oviparous reproductive modes. The data show that Aqp12L silencing disrupts oocyte growth, leading to a developmental shift in the stage-frequency profiles of oocytes in the ovary, characterized by a marked reduction in vitellogenic oocytes and an increased proportion of previtellogenic stages. These results resemble those observed in parthenogenetic *Artemia* and other crustaceans following the silencing of the vitellogenin receptor, where reduced vitellogenin uptake by oocytes decreases offspring production [35,36]. Given the specific localization of Aqp12L in the YPM and the disrupted vitellogenic phenotype of females with silenced Aqp12L, our findings indicate that functional Aqp12L in the YPM is required for oocyte growth. These observations suggest that Aqp12L-mediated water and solute (ammonia, H_2_O_2_, urea) permeability of YPs is necessary for the formation of these vesicles, although the specific function of the channel in YPs is yet unknown.

The dsAqp12L-mediated reduction in oocyte growth appears to be associated with the activation of cell death pathways as evidenced by the increased expression levels of caspase-9, which acts as an initiator of the intrinsic apoptotic pathway [37,38], as well as of caspases-3 and -7, which function as downstream executioners responsible for the dismantling of the cell [39]. However, the mechanism by which the absence of Aqp12L in the YPM compromises vitellogenesis and induces follicular atresia remains unknown. In mammals, genetic knockout of AQP12 in mice resulted in a severe pathology following experimentally induced pancreatitis [9]. The authors proposed that AQP12 may play a key role in the formation and maturation of the ZGs in pancreatic acinar cells, whereby reduced AQP12-mediated water flux across ZG may result in the formation of functionally aberrant granules and enlarged exocytosis vesicles (vacuoles). A similar mechanism might apply to our model, where water flux across YPs facilitated by Aqp12L could be required for proper YP formation and maturation. Accordingly, Aqp12L silencing would lead to aberrant YP organization and cellular damage, ultimately activating apoptotic pathways and impairing oocyte development. Nevertheless, further studies are required to fully understand the physiological role of Aqp12L in *A. franciscana* YPs.

In conclusion, our study provides the first direct evidence that an invertebrate Aqp12L ortholog is a functional polytransporter permeable to water, H_2_O_2_, ammonia, and urea, as are the vertebrate orthologs. In addition, using a combination of immunolocalization and functional approaches, we demonstrate that Aqp12L is present in the YPM of *A. franciscana* oocytes and that it plays an essential role during oocyte development. These findings advance our understanding of the biological roles of the unorthodox aquaporins and lay the groundwork for future studies in invertebrates to elucidate the specific function of Aqp12L in YPs and reproductive adaptations.

## Future directions

Building on the novel findings from the present study, future research should focus on investigating the molecular mechanisms directing invertebrate Aqp12L channels to the intracellular YPM, and how water and/or solute transport across the YPM impacts YP formation and oocyte growth. Since the strong negative effect of Aqp12L RNAi on vitellogenesis resembles that of silenced vitellogenin receptors, it is possible that Aqp12L also plays a role during vitellogenin secretion from the fat body. Such an AQP12-mediated secretion role is postulated for ZGs in vertebrate pancreatic acinar cells and should therefore also be investigated to elucidate the essential biological functions of Aqp12L during invertebrate reproduction.

## Materials and methods

### *X. laevis* and *Artemia franciscana* culture

Adult *X. laevis* and commercially available *A. franciscana* dried cysts were purchased from the Centre de Ressources Biologiques Xénopes (University of Rennes, France) and INVE Aquaculture Inc. (ACUAZUL, S.C., Spain), respectively. Details for the maintenance of frogs and the culture of *A. francsicana* are described elsewhere [15,40,41]. Collection of *X. laevis* oocytes was carried out by surgical laparotomy from anesthetized females with tricaine mesylate (2 g/l in a volume of 1 l of water) following a protocol approved by the Ethics Committee for Animal and Human Experimentation (CEEAH) from UAB and the Catalan Government (Direcció General de Polítiques Ambientals i Medi Natural; Project no. 10985). Frogs were euthanized after three independent surgeries by 2 h immersion in tricaine methanesulfonate (MS222, 5 g/l in 1 l) followed by insertion of a needle through the foramen magnum at the level of the atlanto-occipital joint. All the experiments with animals were carried out at the Institute of Biotechnology and Biomedicine from Universitat Autònoma de Barcelona (Barcelona, Spain).

### Cloning of *A. franciscana* AQP12L and functional characterization

Full-length cDNA encoding *A. franciscana* Aqp12L was isolated by reverse transcription-PCR using cyst total RNA and oligonucleotide primers (Supplementary Table S1) designed based on the *A. franciscana* transcriptome (GenBank accession no. PQ469255) [15], as described previously [41]. The cDNA was fused with the HA epitope tag at the C-terminus of the encoded protein by PCR using specific primers (Supplementary Table S1) and subcloned into the pT7Ts expression vector. The corresponding cRNAs were expressed in *X. laevis* oocytes as described previously [40]. Oocytes were injected with 1 ng of Aqp12L cRNA or left uninjected as controls. As positive controls, oocytes were injected with 15 ng of cRNAs coding for Glp4_v2, EglpL, Aqp7, Aqp7-12CT, or AQP1-12CT. The frog oocyte YPs were isolated by sequential centrifugations of oocyte homogenates in 0.08 M Tris as described previously [11].

Osmotic and solute uptake assays in oocytes and isolated YPs were carried out as described previously [11]. Briefly, the *P*_f_ and *P*_gly_ of oocytes were determined by measuring the volume changes of uninjected oocytes and aquaporin-expressing oocytes in 10-fold diluted Modified Barth’s Solution (MBS: 88 mM NaCl, 1 mM KCl, 2.4 mM, NaHCO_3_, 0.82 mM MgSO_4_, 0.33 mM Ca(NO_3_)_2_, 0.41 mM CaCl2, 10 mM HEPES, and 25 μg/ml gentamycin, pH 7.5), or in MBS containing 160 mM glycerol instead of NaCl. Oocyte permeability to urea, MeNH_2_, and H_2_O_2_ was determined using radiolabeled (urea and MeNH_2_) or fluorescent (H_2_O_2_) compounds. For YPs, water and solute permeabilities were measured also using the same radiolabeled or fluorescent compounds.

### Immunoblotting and immunofluorescence microscopy of *X. laevis* oocytes and YPs

The isolation of the total membrane fraction of *X. laevis* oocytes, YPs, and YPM and immunoblotting were carried out as described previously [11]. Membranes were incubated (1:1000) with HA antibodies (Invitrogen, #PA1-985) overnight at 4°C and in parallel (1:1000) with anti-PDI antibody produced in rabbit (Sigma–Aldrich, #P7496) as control. Both membranes were then incubated (1:5000) with horseradish peroxidase-conjugated goat anti-rabbit IgG secondary antibodies (Bio-Rad, #172-1019) for 1 h at room temperature. Immunoreactive bands were revealed by the Immobilon™ Western chemiluminescent HRP substrate (Merck, #WBKLS) using X-ray films (FUJI RX, #47410-19289).

Immunofluorescence microscopy of oocytes and YPs was carried out as described previously [11]. The slides carrying sections or intact YPs were incubated with anti-HA (1:400) and anti-vitellogenin (Biosense Laboratories, #V01407101) (1:100) antibodies overnight at 4°C in a humidified chamber. Subsequently, the slides were washed three times with PBS and incubated (1:800) with sheep anti-rabbit IgG coupled with Cy3 (Merck, #C2306) for 1 h at room temperature. The slides were washed with PBS and further incubated (1:10 000) with WGA Alexa Fluor^®^ 647 conjugate (Life Technologies Corp., #W32466) for 10 min and washed with PBS. The slides were finally mounted with fluoromount aqueous anti-fading medium (Merck, #F4680), and images were acquired with a Zeiss Axio Imager Z1/ApoTome fluorescence microscope (CarlZeiss Corp., Oberkochen, Germany).

### RNA isolation, cDNA synthesis, and RT-qPCR

Females were anesthetized by immersion in 4 g/l MS222 (in 10 ml) and euthanised by a transfer to a solution of 70% ethanol (in 10 ml). Samples of genital segments containing the gonads (ovary plus ovisac) destined to produce nauplii (ovoviviparous development) or diapause cysts (oviparous development) were subsequently dissected with watchmaker forceps under a stereomicroscope. Free nauplii and diapause cysts were collected from the tanks. The total RNA extraction and reverse transcription were carried out as described previously [15] using specific oligonucleotide primers (Supplementary Table S1). Copy numbers were determined from Ct values and normalized against *A. franciscana* α-tubulin.

### RNAi of Aqp12L in oviparous and ovoviviparous females

Gene knockdown of *aqp12L* was carried out by double-stranded RNA (dsRNA) mediated-RNAi. The MEGAscript^®^ RNAi kit (Invitrogen, #AM1626) was used for the synthesis and purification of dsRNAs following the single PCR protocol described previously [15]. The dsRNAs were prepared using specific forward and reverse oligonucleotide primers (Supplementary Table S1). A total of 100 ng of dsAqp12L RNA were injected into the ovisac of oviparous or ovoviviparous females in a volume of 200 nl of water containing 1% phenol red as described previously [15]. As controls, females were injected with 100 ng of *Aequorea victoria* green fluorescent protein (GFP) dsRNA. Seven days after dsRNA injection, free-swimming nauplii and cysts were collected from the tanks and counted under a stereomicroscope as described previously [15]. Females were euthanized as indicated above to collect the gonads.

### Western blot of *A. franciscana* gonads

The total membrane fractions of gonads, nauplii, and cysts were extracted as for *X. laevis* oocytes, except that after the last centrifugation the pellet was resuspended in 300 μl of *Artemia* lysis buffer (150 mM NaCl, 50 mM Tris pH 8, 1 mM EDTA, 0.5% SDS, 1% Triton X, 40 mM DTT) and sonicated at 20% for 5 min. Immunoblotting was carried out as described above using a custom-made affinity-purified *A. franciscana* Aqp12L rabbit antibody raised against a C-terminus peptide of the channel (GenScript, U.S.A.) (Supplementary Figure S1) at 1:500 dilution, or using the Aqp12L antibody previously preadsorbed with five-fold excess of the immunizing peptide for 1 h at 37°C. Duplicated gels were stained with PlusOne Coomassie Blue PhastGel R-350 (GE Healthcare, Barcelona, Spain) as described previously [42] to ensure equal protein loading.

### Histological analysis

Samples of ovaries were fixed in 4% PFA overnight at 4°C, processed for paraffin sectioning, and stained with hematoxylin and eosin as described previously [29]. Sections were mounted with Eukitt quick-hardening mounting medium (Sigma–Aldrich, #03989), and images were acquired with an Eclipse 90i microscope equipped with a Nikon DS-Ri2 camera (Nikon, Tokio, Japan). To quantify previtellogenic and vitellogenic oocytes, hematoxylin and eosin-stained sections were analyzed from females collected at 3 and 5 days post-dsRNA injection. The number of oocytes was scored in four non-consecutive histological sections per female (6 to 9 females per group).

### Immunofluorescence microscopy

Samples of gonads and cysts were fixed and processed as above. Sections were blocked with PBST (PBS plus 0.05% Tween-20) containing 5% normal goat serum and 0.1% BSA for 1 h at room temperature, and incubated (1:400) overnight at 4°C in a humidified chamber with the *A. franciscana* Aqp12L antibody. Sections were subsequently washed three times with PBS and incubated (1:800) with sheep anti-rabbit IgG coupled with Cy3 (Merck, #C2306) for 1 h at room temperature. Control sections were incubated with the Aqp12L antibody previously preadsorbed as indicated above. Subsequently, the sections were washed with PBS and further incubated (1:10 000) with WGA for 10 min and washed with PBS. The sections were mounted with fluoromount aqueous anti-fading medium (Merck, #F4680), and images were acquired with a Zeiss Axio Imager Z1/ApoTome fluorescence microscope (CarlZeiss Corp., Oberkochen, Germany).

### Determination of caspase-3/7 and caspase-9 activity

Gonad samples from dsGFP RNA- and dsAqp12L RNA-injected females (10 per group) were collected at day 3 and 5 after dsRNA treatment. Caspase activity was measured following a similar protocol as that described by Menze and Hand [43]. Samples were homogenized in 500 μl of buffer containing 0.5 M sucrose, 20 mM HEPES, and 10 mM DTT with a manual pestle and centrifuged at 14 000 × ***g*** for 15 min at 4°C. The supernatant was transferred to fresh tubes, and total protein concentration was determined by the Pierce BCA protein assay kit (ThermoScientific, #23227) following the manufacturer’s instructions. Caspase-3/7 and caspase-9 assays were run with 1 mg/ml of total protein using the (Z-DEVD)2-R110 (ATT Bioquest, #13430) and Z-LEHD-ProRed™ 620 (ATT Bioquest, #13435) kits, respectively, following manufacturer’s instructions. The fluorescence was measured using Spark^®^ multimode microplate reader (Tecan Trading AG, Männedorf, Switzerland) after 1 h of incubation at room temperature protected from light.

### Statistics

The GraphPad Prism v9.4.1 software (GraphPad Software) was used for statistical analyses. Unpaired Student’s *t*-test and one-way analysis of variance (ANOVA), followed by the Tukey’s multiple comparison test, were used for the different experiments as stated in the figure legends. Error bars represent SEM. In all cases, statistical significance was defined as *P* < 0.05 (*), *P* < 0.01 (**), or *P* < 0.001 (***).

## Supplementary Material

Supplementary Figures S1-S5 and Table S1

## Data Availability

The authors confirm that the data supporting the findings of the present study are available within the article and its supplementary material.

## Open Access

Open access for this article was enabled by the participation of Institut de Ciencies del Mar in an all-inclusive Read & Publish agreement with Portland Press and the Biochemical Society under an agreement with CSIC.

## Abbreviations

Aqp12L: AQP12-like
AQP/Aqp: aquaporin
BSA: bovine serum albumin
BF: bright field
cDNA: complementary DNA
CDS: coding sequence
DAPI: 4′,6-diamidino-2-phenylindole
DPI: days post injection
dsRNA: double-stranded RNA
EglpL: entomoglyceroporin-lke
ER: endoplasmic reticulum
GFP: green fluorescent protein
Glp4_v2: aquaglyceroporin-4 isoform 2
HA: human influenza hemagglutinin
LEA: late embryogenesis abundant
PBS: phosphate-buffered saline
PDI: protein disulfide-isomerase
*P*_f_: osmotic water permeability
*P*_gly_: osmotic glycerol permeability
RNAi: RNA interference
RT-qPCR: quantitative reverse transcription polymerase chain reaction
WGA: wheat germ agglutinin
YP: yolk platelet
YPM: yolk platelet membrane
ZG: zymogen granules

## References

[1] King L.S., Kozono D. and Agre P. (2004) From structure to disease: the evolving tale of aquaporin biology. Nat. Rev. Mol. Cell Biol. 5, 687–698 15340377 10.1038/nrm1469

[2] Bienert G.P. and Chaumont F. (2014) Aquaporin-facilitated transmembrane diffusion of hydrogen peroxide. Biochim. Biophys. Acta 1840, 1596–1604 24060746 10.1016/j.bbagen.2013.09.017

[3] Finn R.N. and Cerdà J. (2015) Evolution and functional diversity of aquaporins. Biol. Bull. 229, 6–23 26338866 10.1086/BBLv229n1p6

[4] Abascal F., Irisarri I. and Zardoya R. (2014) Diversity and evolution of membrane intrinsic proteins. Biochim. Biophys. Acta 1840, 1468–1481 24355433 10.1016/j.bbagen.2013.12.001

[5] Finn R.N., Chauvigné F., Hlidberg J.B., Cutler C.P. and Cerdà J. (2014) The lineage-specific evolution of aquaporin gene clusters facilitated tetrapod terrestrial adaptation. PloS ONE 9, e113686 25426855 10.1371/journal.pone.0113686 PMC4245216

[6] Stavang J.A., Chauvigné F., Kongshaug H., Cerdà J., Nilsen F. and Finn R.N. (2015) Phylogenomic and functional analyses of salmon lice aquaporins uncover the molecular diversity of the superfamily in *Arthropoda*. BMC Genomics 16, 618 26282991 10.1186/s12864-015-1814-8 PMC4539701

[7] Irisarri I., Lorente-Martínez H., Strassert J.F., Agorreta A., Zardoya R., San Mauro D. et al. (2024) Early diversification of membrane intrinsic proteins (MIPs) in eukaryotes. Genome Biol. Evol. 16, evae164 39058319 10.1093/gbe/evae164 PMC11316224

[8] Itoh T., Rai T., Kuwahara M., Ko S.B., Uchida S., Sasaki S. et al. (2005) Identification of a novel aquaporin, AQP12, expressed in pancreatic acinar cells. Biochem. Biophys. Res. Commun. 330, 832–838 15809071 10.1016/j.bbrc.2005.03.046

[9] Ohta E., Itoh T., Nemoto T., Kumagai J., Ko S.B., Ishibashi K. et al. (2009) Pancreas-specific aquaporin 12 null mice showed increased susceptibility to caerulein-induced acute pancreatitis. Am. J. Physiol. Cell Physiol. 297, C1368–C1378 19726746 10.1152/ajpcell.00117.2009

[10] Ishibashi K., Tanaka Y. and Morishita Y. (2021) The role of mammalian superaquaporins inside the cell: an update. Biochim. Biophys. Acta Biomembr. 1863, 183617 33811846 10.1016/j.bbamem.2021.183617

[11] Chauvigné F., Catalán-García M., Daura X., Finn R.N. and Cerdà J. (2026) A pan-vertebrate signaling motif controls the molecular function of intracellular AQP12. J. Cell Biol. 225, e202512040 42390377 10.1083/jcb.202512040 PMC13344155

[12] Wallace R.A. and Selman K. (1990) Ultrastructural aspects of oogenesis and oocyte growth in fish and amphibians. J. Electron Microsc. Tech. 16, 175–201 2243277 10.1002/jemt.1060160302

[13] Ramos I., Machado E., Masuda H. and Gomes F. (2022) Open questions on the functional biology of the yolk granules during embryo development. Mol. Reprod. Dev 89, 86–94 35020238 10.1002/mrd.23555

[14] Wang W., Zhang X.S., Wang Z.N. and Zhang D.X. (2023) Evolution and phylogenetic diversity of the aquaporin gene family in arachnids. Int. J. Biol. Macromol. 240, 124480 37068537 10.1016/j.ijbiomac.2023.124480

[15] Rey-Alfonso Á., Maestro J.L., Chauvigné F., Gómez-Garrido J., Alioto T., Bossier P. et al. (2025) Co-opted and canonical glycerol channels play a major role during anhydrobiosis of an extremophile crustacean. BMC Biol. 23, 151 10.1186/s12915-025-02262-3 PMC12135271 40462055

[16] Zhang L., Pang Y., Yang H. and Yang Z. (2023) Cloning and characterization of aquaporins 11 and its expression analysis under molt cycle in *Eriocheir sinensis*. Int. J. Aquaculture 13, 1–9 10.5376/ija.2023.13.0001

[17] Wang Z., Song G., Zhang S., Zhang L., Wang B., Zhao K. et al. (2025) Silencing of the superaquaporin LvAQP11 disrupts salinity tolerance, molting cycle, and myofibril organization in *Litopenaeus vannamei*. Fishes 10, 631 10.3390/fishes10120631

[18] Lind U., Järvå M., Alm Rosenblad M., Pingitore P., Karlsson E., Wrange A.L. et al. (2017) Analysis of aquaporins from the euryhaline barnacle *Balanus improvisus* reveals differential expression in response to changes in salinity. PloS ONE 12, e0181192 28715506 10.1371/journal.pone.0181192 PMC5513457

[19] Abatzopoulos T.J., Beardmore J.A., Clegg J.S. and Sorgeloos P.(eds). (2002) Artemia: Basic and Applied Biology, Kluwer Academic Publishers, Dordrecht

[20] Nambu Z., Tanaka S. and Nambu F. (2004) Influence of photoperiod and temperature on reproductive mode in the brine shrimp, *Artemia franciscana*. J. Exp. Zool. A Comp. Exp. Biol. 301, 542–546 15181648 10.1002/jez.a.80

[21] Hand S.C., Denlinger D.L., Podrabsky J.E. and Roy R. (2016) Mechanisms of animal diapause: recent developments from nematodes, crustaceans, insects, and fish. Am. J. Physiol. Regul. Integr. Comp. Physiol. 310, R1193–R1211 27053646 10.1152/ajpregu.00250.2015 PMC4935499

[22] Chauvigné F., Lubzens E. and Cerdà J. (2011) Design and characterization of genetically engineered zebrafish aquaporin-3 mutants highly permeable to the cryoprotectant ethylene glycol. BMC Biotechnol. 11, 34 21477270 10.1186/1472-6750-11-34 PMC3079631

[23] Clegg J.S. (1964) The control of emergence and metabolism by external osmotic pressure and the role of free glycerol in developing cysts of *Artemia Salina*. J. Exp. Biol. 41, 879–892 14239915 10.1242/jeb.41.4.879

[24] Clegg J.S. (1965) The origin of trehalose and its significance during the formation of encysted dormant embryos of *Artemia*. Comp. Biochem. Physiol. 14, 135–143 14288194 10.1016/0010-406X(65)90014-9

[25] Liang P. and MacRae T.H. (1999) The synthesis of a small heat shock/alpha-crystallin protein in *Artemia* and its relationship to stress tolerance during development. Dev. Biol. 207, 445–456 10068475 10.1006/dbio.1998.9138

[26] Chen T., Villeneuve T.S., Garant K.A., Amons R. and MacRae T.H. (2007) Functional characterization of artemin, a ferritin homolog synthesized in *Artemia* embryos during encystment and diapause. FEBS J. 274, 1093–1101 17257268 10.1111/j.1742-4658.2007.05659.x

[27] Hand S.C., Jones D., Menze M.A. and Witt T.L. (2007) Life without water: expression of plant LEA genes by an anhydrobiotic arthropod. J. Exp. Zool. A Ecol. Genet. Physiol. 307, 62–66 17109393 10.1002/jez.a.343

[28] Liu Y.L., Zhao Y., Dai Z.M., Chen H.M. and Yang W.J. (2009) Formation of diapause cyst shell in brine shrimp, *Artemia parthenogenetica*, and its resistance role in environmental stresses. J. Biol. Chem. 284, 16931–16938 19395704 10.1074/jbc.M109.004051 PMC2719330

[29] Rey-Alfonso Á., Chauvigné F., Finn R.N. and Cerdà J. (2025) New insights into the anhydrobiotic role of trehalose biosynthesis and transport in *Artemia franciscana* diapause embryos. Open Biol 15, 250176 10.1098/rsob.250176 41537825

[30] MacRae T.H. (2016) Stress tolerance during diapause and quiescence of the brine shrimp. *Artemia*. Cell Stress Chaperones 21, 9–18 26334984 10.1007/s12192-015-0635-7 PMC4679736

[31] King A.M., Toxopeus J. and MacRae T.H. (2014) Artemin, a diapause-specific chaperone, contributes to the stress tolerance of *Artemia franciscana* cysts and influences their release from females. J. Exp. Biol. 217, 1719–17124 24526727 10.1242/jeb.100081

[32] Toxopeus J., Warner A.H. and MacRae T.H. (2014) Group 1 LEA proteins contribute to the desiccation and freeze tolerance of *Artemia franciscana* embryos during diapause. Cell Stress Chaperones 19, 939–948 24846336 10.1007/s12192-014-0518-3 PMC4389855

[33] Rowarth N.M. and MacRae T.H. (2018) ArHsp40 and ArHsp40-2 contribute to stress tolerance and longevity in *Artemia franciscana*, but only ArHsp40 influences diapause entry. J. Exp. Biol. 221, jeb189001 30158133 10.1242/jeb.189001

[34] Iryani M.T.M., Sorgeloos P., Danish-Daniel M., Tan M.P., Wong L.L., Mok W.J. et al. (2020) Cyst viability and stress tolerance upon heat shock protein 70 knockdown in the brine shrimp *Artemia franciscana*. Cell Stress Chaperones 25, 1099–1103 32383141 10.1007/s12192-020-01113-0 PMC7591639

[35] Ruan Y., Wong N.K., Zhang X., Zhu C., Wu X., Ren C. et al. (2020) Vitellogenin receptor (VgR) mediates oocyte maturation and ovarian development in the Pacific white shrimp (*Litopenaeus vannamei*). Front. Physiol. 11, 485 32499719 10.3389/fphys.2020.00485 PMC7243368

[36] Duan H., Shao X., Liu W., Xiang J., Pan N., Wang X. et al. (2023) Spatio-temporal patterns of ovarian development and VgR gene silencing reduced fecundity in parthenogenetic *Artemia*. Open Biol. 13, 230172 37963545 10.1098/rsob.230172 PMC10645507

[37] Li P., Nijhawan D., Budihardjo I., Srinivasula S.M., Ahmad M., Alnemri E.S. et al. (1997) Cytochrome c and dATP-dependent formation of Apaf-1/caspase-9 complex initiates an apoptotic protease cascade. Cell 91, 479–489 9390557 10.1016/S0092-8674(00)80434-1

[38] Zou H., Li Y., Liu X. and Wang X. (1999) An APAF-1.cytochrome c multimeric complex is a functional apoptosome that activates procaspase-9. J. Biol. Chem. 274, 11549–11556 10206961 10.1074/jbc.274.17.11549

[39] Lakhani S.A., Masud A., Kuida K., Porter G.A. Jr, Booth C.J., Mehal W.Z. et al. (2006) Caspases 3 and 7: key mediators of mitochondrial events of apoptosis. Science 311, 847–851 16469926 10.1126/science.1115035 PMC3738210

[40] Chauvigné F., Ferré A. and Cerdà J. (2021) The *Xenopus* oocyte as an expression system for functional analyses of fish aquaporins. Methods Mol. Biol. 2218, 11–28 33606219 10.1007/978-1-0716-0970-5_2

[41] Ferré A., Chauvigné F., Vlasova A., Norberg B., Bargelloni L., Guigó R. et al. (2023) Functional evolution of clustered aquaporin genes reveals insights into the oceanic success of teleost eggs. Mol. Biol. Evol. 40, msad071 36947084 10.1093/molbev/msad071 PMC10075191

[42] Zapater C., Chauvigné F., Norberg B., Finn R.N. and Cerdà J. (2011) Dual neofunctionalization of a rapidly evolving aquaporin-1 paralog resulted in constrained and relaxed traits controlling channel function during meiosis resumption in teleosts. Mol. Biol. Evol. 28, 3151–3169 21653921 10.1093/molbev/msr146

[43] Menze M.A. and Hand S.C. (2007) Caspase activity during cell stasis: avoidance of apoptosis in an invertebrate extremophile, *Artemia franciscana*. Am. J. Physiol. Regul. Integr. Comp. Physiol. 292, R2039–R2047 17255212 10.1152/ajpregu.00659.2006

