# Supplementary material for: The unorthodox aquaporin-12L ortholog is essential for oogenesis in the crustacean Artemia franciscana
Source: Biosci Rep. 2026 Oct 13;46(10):BSR20260496. doi: 10.1042/BSR20260496 (PMC13673172; doi:10.1042/BSR20260496)
Supplement: Supplementary file 1 [file BSR-2026-0496_supp.pdf]

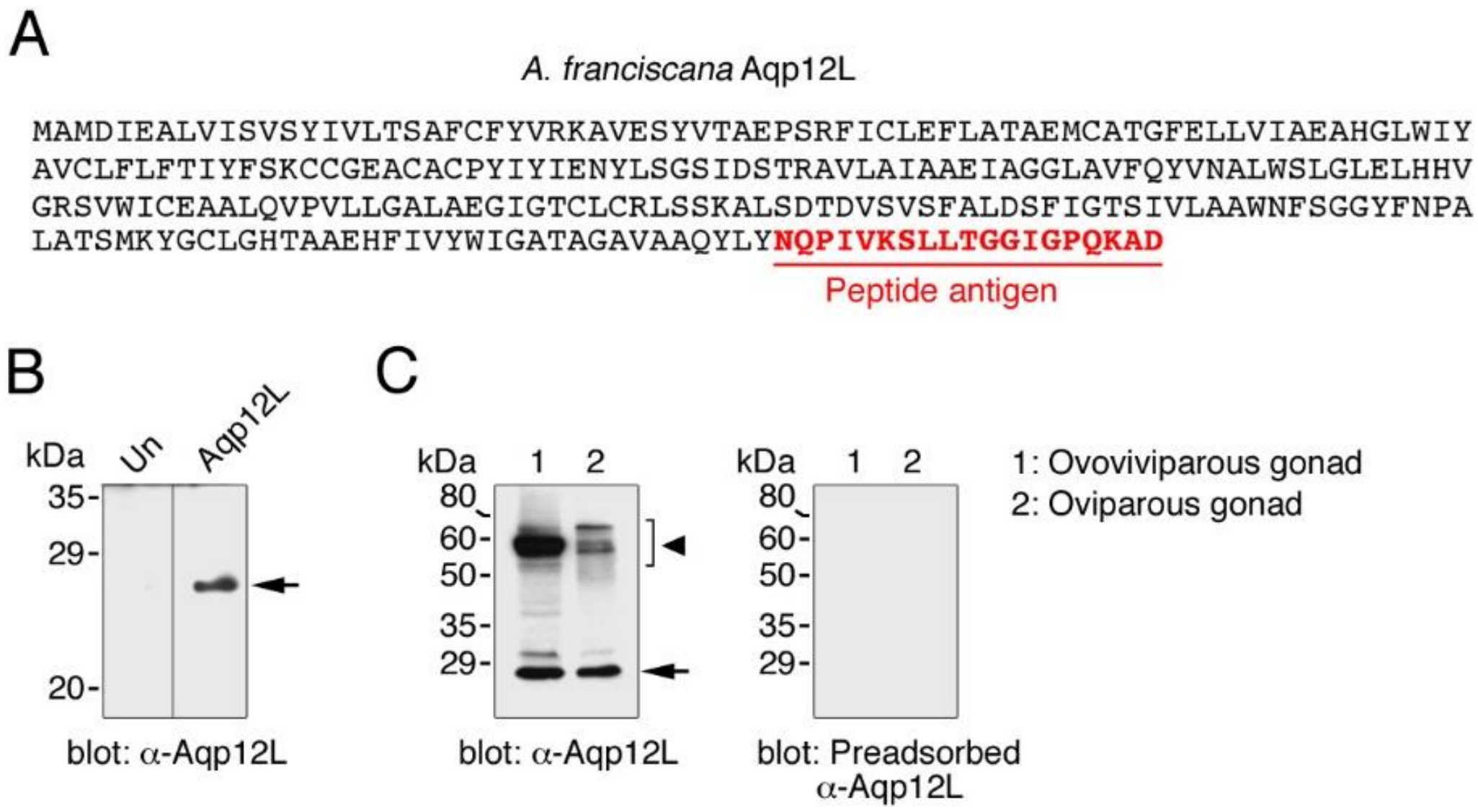

Figure S1: Characterization of a custom-made rabbit antibody raised against *A. franciscana* Aqp12L (α-Aqp12L)

(A) Amino acid sequence of *A. franciscana* Aqp12L showing the peptide antigen used for rabbit immunization. (B) Western blot of protein extracts from *X. laevis* uninjected (Un) oocytes and oocytes expressing Aqp12L using the α-Aqp12L antibody. (C) Immunoblot of *A. franciscana* ovoviviparous and oviparous ovisacs (left) probed with the antibody. A duplicate blot that was run in parallel and incubated with the preabsorbed antisera (right). The arrows in the blots shown in B and C point to the immunoreactive bands that closely match the predicted monomeric molecular mass of Aqp12L (28.4 kDa), whereas the arrowhead indicates potential post-translational modifications and/or oligomerization of the channel. Molecular markers (kDa) are on the left. Uncropped blots are shown in Supplementary Figure S5.

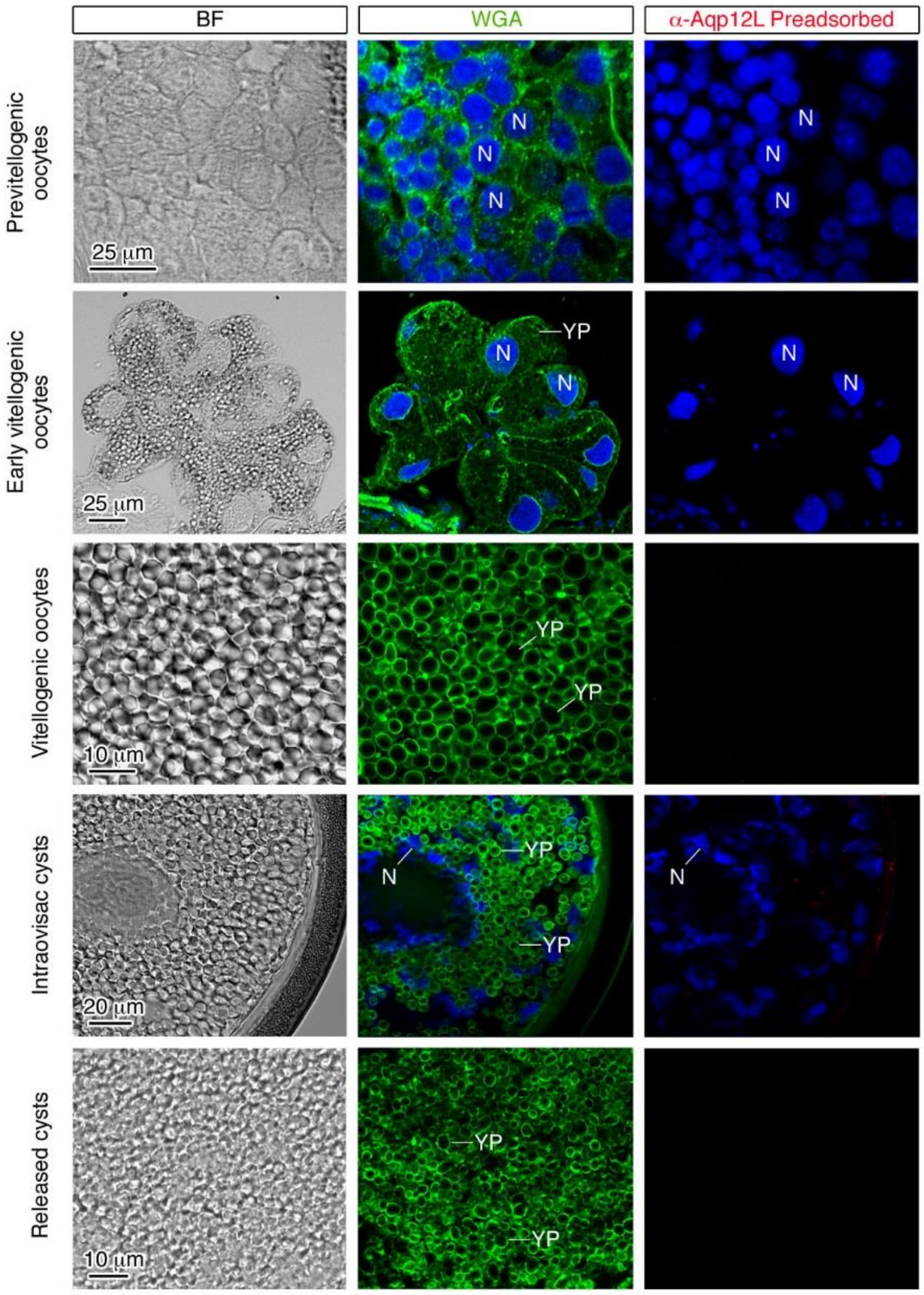

Figure S2: Immunostaining controls of the localization of Aqp12L during *A. franciscana* ovoviviparous and oviparous development

Histological sections were incubated with the Aqp12L antiserum preabsorbed with the immunizing peptide, indicating the specificity of the antibodies. N, nuclei; YP, yolk platelet,

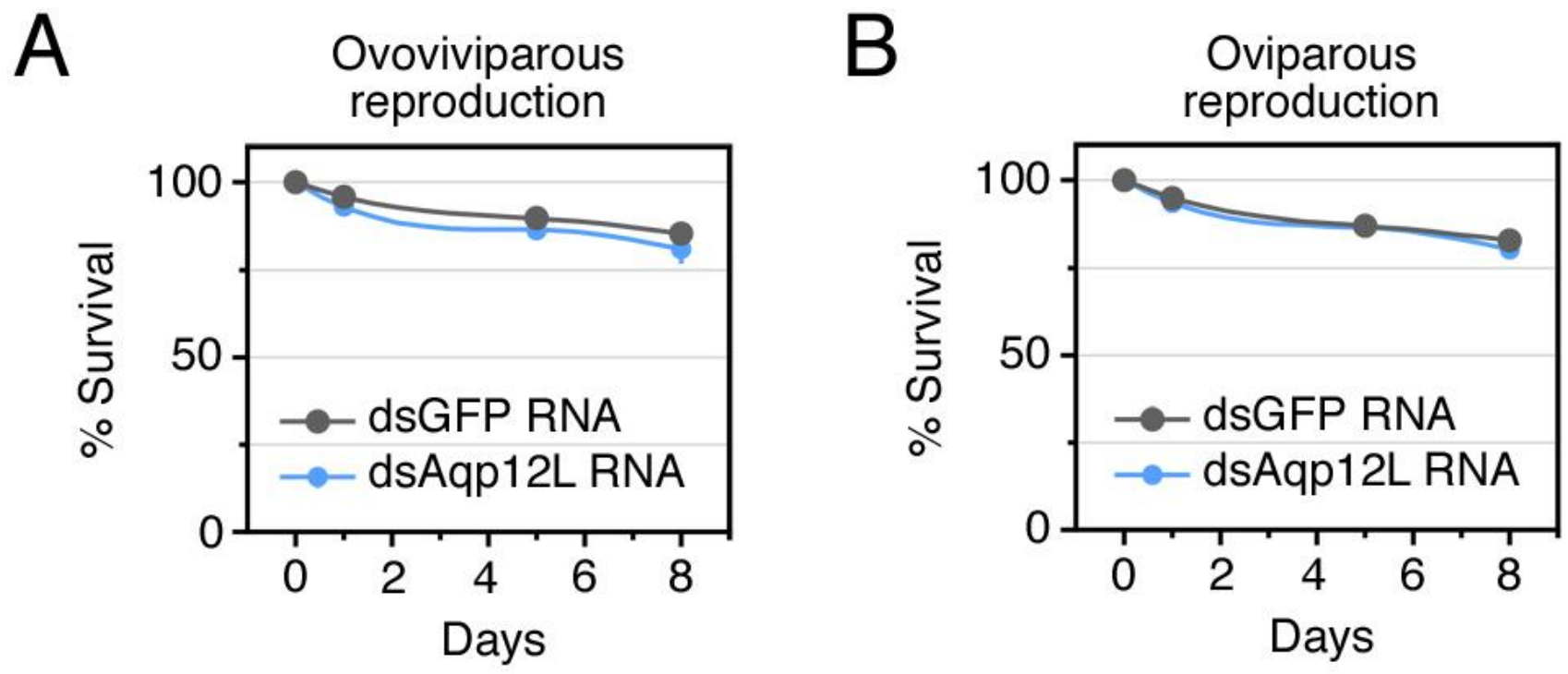

Figure S3: Survival of females injected with dsGFP (control) or dsAqp12L RNAs during ovoviviparous and oviparous reproduction.

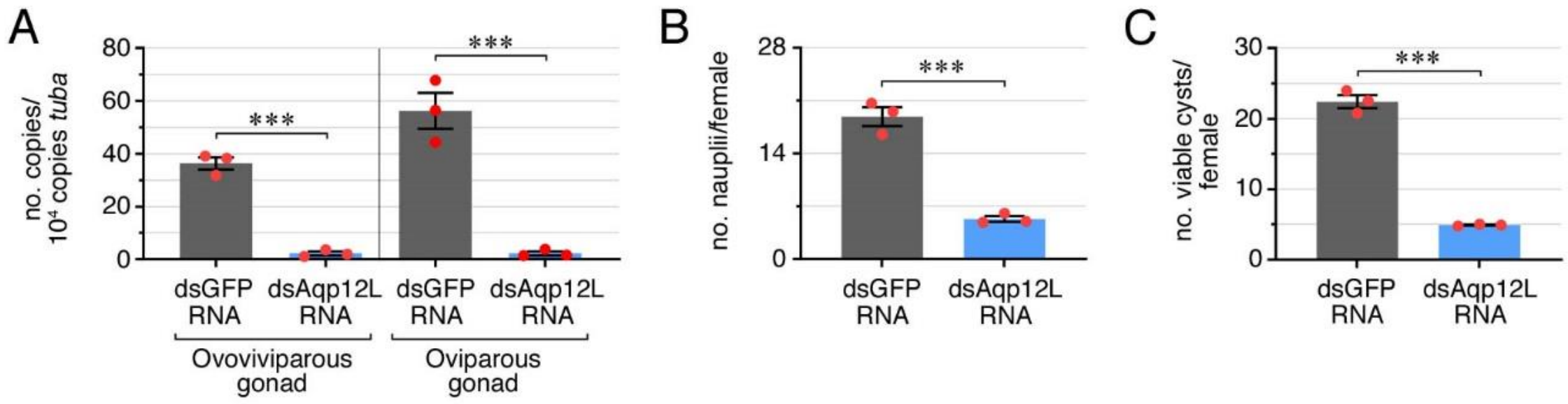

Figure S4: Effects of RNAi-mediated Aqp12L knockdown on ovoviviparous and oviparous development using a second dsRNA

(A) Decrease of *aqp12L* transcript levels in gonads (ovary and ovisac) from ovoviviparous (A) or oviparous (D) females injected with dsGFP (control) or dsAqp12L RNAs determined by RT-qPCR. (B-C) Production of nauplii (C) or diapause cysts (F) by females treated with dsGFP or dsAqp12L RNAs. In each panel, data (mean ± SEM; n = 3 independent experiments, red dots) were statistically analyzed by an unpaired Student's t-test (***, $P < 0.001$; with respect to the controls).

Figure 1C

Aqp12L

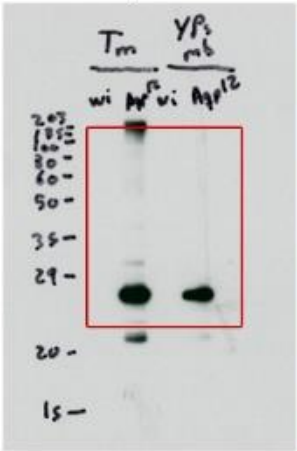

Pdi

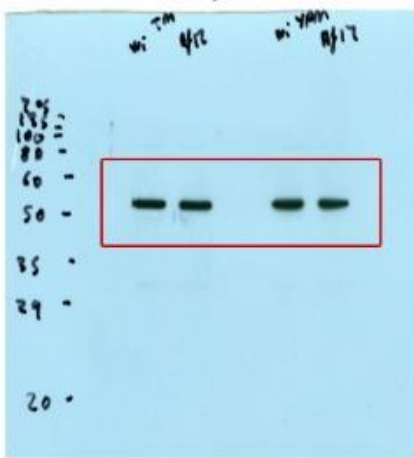

Figure 2B

Coomassie Blue

Aqp12L

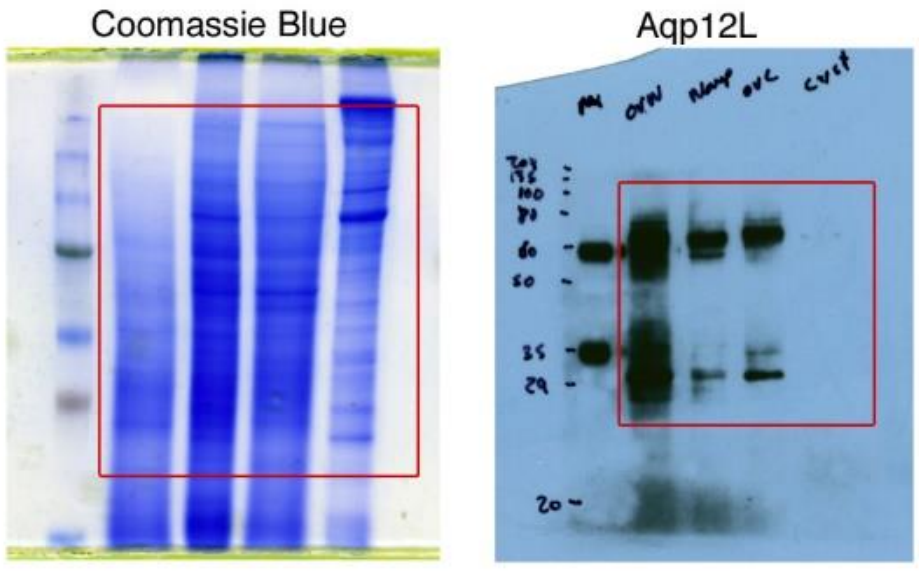

Figure 3B

Coomassie Blue

Aqp12L

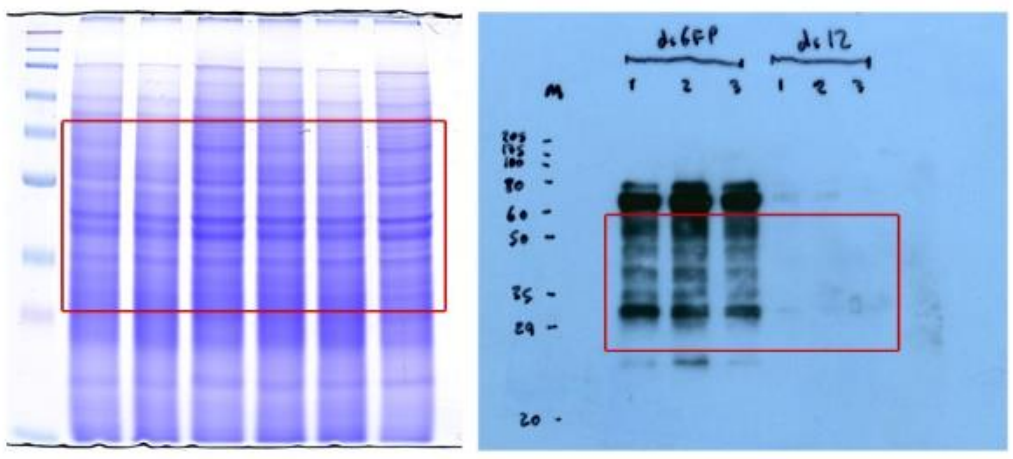

Figure 3E

Coomassie Blue

Aqp12L

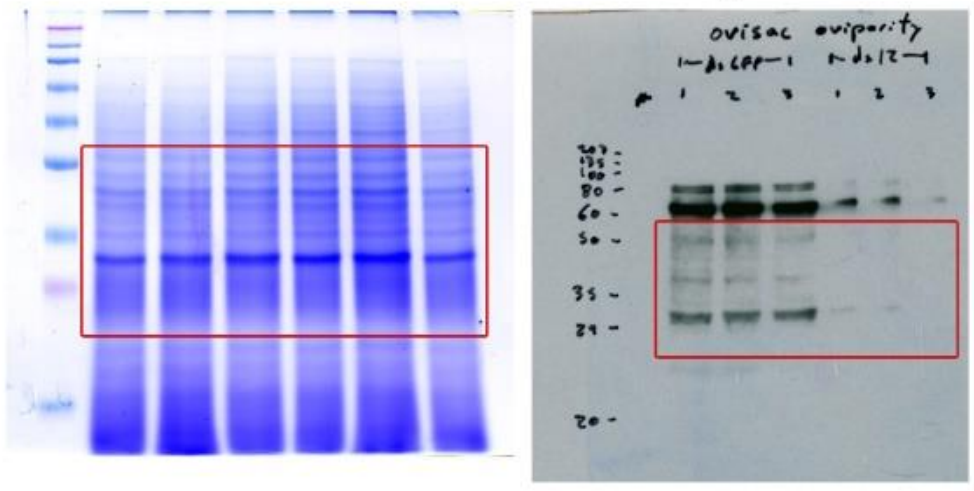

Figure S1B

Aqp12L Xenopus oocytes

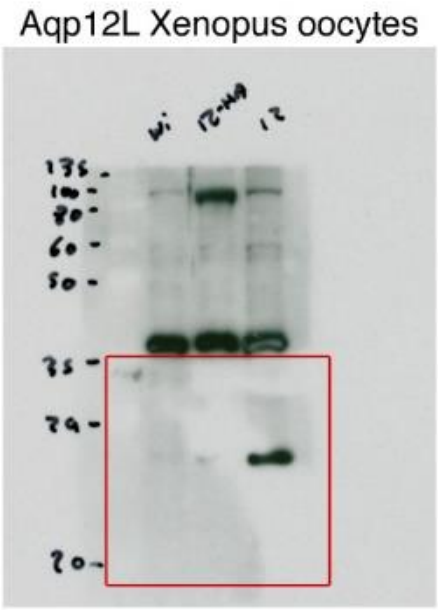

Figure S1C

Aqp12L stages

Preincubated

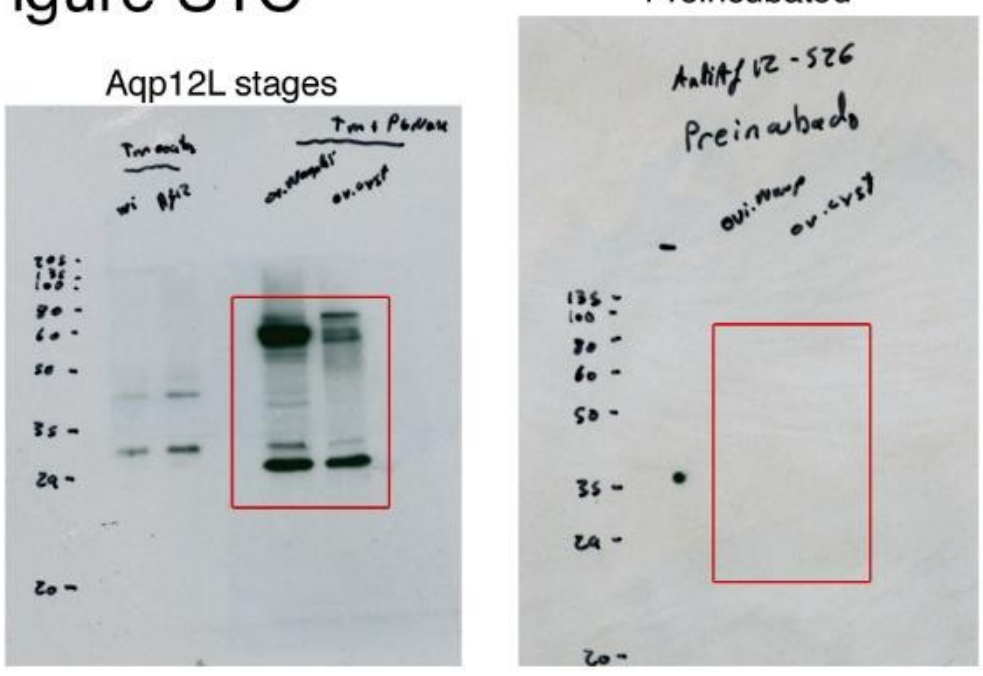

Figure S5: Uncropped Coomassie Blue images corresponding to Figure 2B, 3B and 3E, and Western blot images corresponding to Figure 1C, 2B, 3B, 3E, S1B and S1C.

Supplementary Table S1. Oligonucleotide primers used for cloning, RT-qPCR and synthesis of dsRNAs

Gene/dsRNA	Purpose	Primer sequence Forward-Reverse (5'-3')	Amplicon size (bp)
Aqp12L	Cloning	F: TCATTCCAGTCTGCCAAAAA<br>R: TCTGGTTAGGCTGTATTGTAGCA	951
Aqp12L-HA [1]	Cloning	F: GCGATATCTCATTCCAGTCTGCCAAAAA<br>R: CGACTAGTTTAAGCGTAATCTGGAACATCGTATGGGTAGTCCGCTTTCTGAGGACCTA	978
α-Tubulin	RT-qPCR	F: CCCGTCTTGACCACAAGTTT<br>R: TAATACTCTTCGCCGGCTTC	187
Aqp12L	RT-qPCR	F: CCTTGGCCACCTCTATGAAG<br>R: GTCCGCTTTCTGAGGACCTA	167
dsGFP [2]	RNAi	F: TAATACGACTCACTATAGGGAGAGCCCTGGCCCACCCT<br>R: TAATACGACTCACTATAGGGAGACAGCACGGGGCCGTC	418
dsAqp12L (nt 270-691) [2]	RNAi	F: TAATACGACTCACTATAGGGAGACTGTCCTTATATCTA<br>R: TAATACGACTCACTATAGGGAGAATACGATGAAGTGTT	422
dsAqp12L (nt 6-268) [2]	RNAi	F: TAATACGACTCACTATAGGGAGACATGGACATTGAAGC<br>R: TAATACGACTCACTATAGGGAGACACAGGCTTCACCAC	263

[1] The nucleotide sequence coding for the HA tag amino acid sequence (YPYDVPDYA) is double underlined.

[2] The T7 promoter sequence added to each primer is underlined. The dsGFP RNA was synthesized from the pEGFP1 plasmid (Addgene; https://www.addgene.org/vector-database/2486/
